# Combo Deals, Junk Meals: A Systematic Review Examining the Healthiness of Foods Promoted on Meal Delivery Apps

**DOI:** 10.1111/obr.70097

**Published:** 2026-02-09

**Authors:** Jessica Morrison, Joanne Dono, Simone Pettigrew, Caroline Miller

**Affiliations:** ^1^ Adelaide University School of Public Health Adelaide South Australia Australia; ^2^ Health Policy Centre, South Australian Health & Medical Research Institute (SAHMRI), North Tce Adelaide South Australia Australia; ^3^ Adelaide University School of Psychology Adelaide South Australia Australia; ^4^ The George Institute for Global Health The University of New South Wales Sydney New South Wales Australia

**Keywords:** food promotion, meal delivery apps, nutrition policy, online food delivery

## Abstract

**Introduction:**

Meal delivery apps increase access to unhealthy foods, contributing to an obesogenic environment. Promotions are widely used on these apps to influence consumer choice. This systematic review aimed to synthesize evidence on the healthiness of foods promoted on meal delivery apps.

**Methods:**

A systematic search for studies published from 2010 to November 2024 was conducted using three electronic academic databases and two search engines. Studies were included if they assessed promotion on third‐party aggregator meal delivery apps and used an objective measure of healthiness.

**Results:**

The search returned 1449 articles, of which nine articles from four countries met the inclusion criteria. Three studies assessed food outlet menus, two assessed individual menu items on app home pages, and five assessed menu items on outlet pages. Most studies found that unhealthy foods featured more prominently than healthy foods through combination deals, volume incentives, and prioritized placement.

**Conclusions:**

Findings from this review indicate that unhealthy menu items offered on meal delivery apps are more likely than healthy menu items to be the subject of promotional strategies. Considerable variation in study methods limited the quantitative integration of findings, highlighting the need for greater methodological consistency in this evolving research field. Building a strong evidence base will facilitate appropriate policy development to support healthier choices on meal delivery apps.

## 1 Introduction

Unhealthy diets, including those with excess intakes of salt, sugars and fats, are one of the leading risk factors for noncommunicable diseases [1]. Globally, dietary risk factors account for 11 million deaths and 255 million disability‐adjusted life years [2]. Worldwide, overweight and obesity affect 43% and 16% of adults, respectively [3]. The obesity epidemic is facilitated by obesogenic food environments [3, 4], characterized by increased accessibility and pervasive marketing of unhealthy foods prepared away from home [5].

An established link exists between increased consumption of foods prepared away from home (e.g., take‐away or restaurants) and increased risk of noncommunicable diseases [6, 7, 8, 9]. Eating foods prepared away from home is associated with increased intakes of energy, saturated fat, and sodium [7]. Foods prepared away from home have become a significant component of many people's diets, contributing 25% of UK adults' daily energy intake [10] and over 40% of Australian young adults' daily energy intake [11]. Furthermore, a five‐country analysis found that over 75% of populations from all included countries consumed foods prepared away from home at least once per week [12].

Technological advances driving innovation in online food delivery through meal delivery apps have facilitated accessibility to outlets selling food prepared away from home [13], contributing to the obesogenic environment. Online food delivery encompasses online grocery stores (e.g., online supermarket), meal kit services (e.g., HelloFresh), and meal delivery apps (MDAs; e.g., UberEats) [3]. MDAs are third‐party digital aggregator platforms that offer consumers a single point of access to multiple food outlets, such as restaurants, cafes, and fast food outlets [13]. The use of MDAs has increased rapidly [5], with an estimated 2.2 billion users globally [6]. A large multicountry study found that the proportion of people who used MDAs at least weekly significantly increased from 19% of the adult population in 2018 to 25% in 2021 [12].

MDAs increase the number and variation of food outlets accessible to an individual compared to their physical environment [14]. The high proportion of unhealthy menu items featured on MDAs may have potential negative impacts on population health [15]. Furthermore, the growing body of international evidence shows that along with convenience and taste, price is a driving factor for consumers when deciding what to order from MDAs [16, 17, 18, 19]. This is concerning, given evidence from other food environments has demonstrated the influence of pricing [20] and prominent placement in encouraging unhealthy food choices [21].

Promotion, a core component of marketing that aims to communicate a product's value to consumers, aims to encourage purchasing of a product through strategies like advertising and price incentives [22]. Varieties of promotional strategies, including prominent outlet placement on the homepage, images of foods, and price‐based offers (e.g., discounts, combination [“combo”] deals), have been observed on MDAs in different countries [23, 24]. Proprietary algorithms are employed by MDAs to personalize the app's interface [25]. A recent systematic scoping review across the entire digital food environment (including grocery, food meal kit, and MDAs) aimed to understand the potential impacts of the digital food environment on population diets and health [15]. The study demonstrated that the offerings available through online delivery platforms were mostly unhealthy [15]. Furthermore, the review included a limited number of studies examining promotions in that setting, which showed that unhealthy menu items were promoted more than healthy menu items [15]. However, a detailed analysis regarding the relationship between specific promotional strategies and healthiness of foods on MDAs is yet to be done. This is an important next step to understanding the potential drivers of food choice on MDAs.

MDAs currently act with limited government oversight, exempt from existing nutrition‐related policies in the out‐of‐home food sector [26, 27]. Existing policies relating to food retail, composition, and pricing have the capacity to include MDAs [28]. Additionally, public health experts have suggested that policies limiting price promotions for unhealthy menu items and increasing those for healthy menu items on MDAs are likely to improve dietary choices [27]. High‐quality evidence is needed to support governments in updating nutrition‐related policy to include MDAs.

Given the rapidly expanding field of research and the importance of understanding the relationship between specific promotional attributes (e.g., prioritized placement; price discounts) and the relative healthiness of foods on MDAs, a systematic review that quantifies these relationships is warranted. The current review aimed to synthesize the data from multiple studies on MDAs that have assessed the nutritional quality of promoted foods. This will provide timely evidence needed to better understand how MDAs are presenting foods to consumers and guide the development of public health policy interventions aimed at improving population‐level food choices [29, 30].

## 2 Methods

This review was conducted in line with the COSMOS‐E guidance for conducting systematic reviews and meta‐analyses of observational studies and reported in line with the Preferred Reporting Items for Systematic Reviews and Meta‐analyses (PRISMA) guidelines, reported in Table S1 [31, 32]. The review protocol was prospectively registered on the International prospective register of systematic reviews (PROSPERO) (ID CRD42023478857).

### 2.1 Inclusion Criteria

The Population, Intervention, Comparison, Outcomes and Study (PICOS) framework used to establish the inclusion criteria that defined the review question is shown in Table 1. Studies that were written in English, assessed third‐party aggregator MDAs (e.g., UberEats), measured the promotion of food on an MDA, and used objective measures of healthiness (e.g., national dietary guidelines) were included. Given the differences in nutritional classifications used across different studies, this review categorized foods as healthy or unhealthy on the basis of classification systems used in included studies (e.g., discretionary items were categorized as unhealthy and core items were categorized as healthy).

**TABLE 1:** Population, Intervention, Comparison, Outcomes and Study (PICOS) framework for eligibility criteria of articles assessing the healthiness of foods available on meal delivery apps.

	Include	Exclude
**Population**	Third‐party aggregator meal delivery app menus and items	Single outlet meal delivery apps, meal delivery kits, meal delivery subscription programs
**Intervention**	Promotional techniques (e.g., “most popular” or “healthy” categories, combination deals, price promotions, images)	Promotion that is external to meal delivery apps (e.g., social media advertising)
**Comparison**	Healthiness (objective measures)	Subjective measures of healthiness
**Outcome**	Primary: relative prevalence of unhealthy food promotions Secondary: characteristics/frequencies of promotions	Health of delivery personnel, environmental impacts (e.g., packaging, transport)
**Study design**	Randomized‐control trial, cross‐sectional studies, cohort studies, case–control studies, objective data from meal delivery apps	Reviews, theses/dissertations, commentaries/editorials, not objective data from meal delivery apps

### 2.2 Search Strategy

An initial literature search was performed on PubMed to explore the breadth of research on the topic and to identify key words. This preliminary search was used to develop the systematic search strategy for PubMed (Table S2). This strategy was adapted for Scopus and Embase to ensure comprehensive retrieval of relevant articles, as these databases index a range of different journals. A broad approach was taken to the search strategy due to the limited number of published articles in this emerging field. A complementary search was undertaken on Google Scholar and Google (screening the top 100 results of each) to capture any government and nongovernment organization (NGO) documents.

The main search was initially conducted in January 2024. The search was restricted to articles from 2010 onward to ensure that the results are relevant to the current landscape of food promotion within MDAs, which emerged in the years following the launch and expansion of “app stores” (e.g., Apple App Store; Google Play) between 2008 and 2012 [33]. An updated search was performed in November 2024. Forward and backward citation searches were conducted on included studies.

### 2.3 Study Selection

All search results were exported to Endnote 20 and imported into Covidence to streamline the systematic review process [34, 35]. Duplicates were removed and an initial review of a sample of articles (10%, *n* = 89) was conducted independently by two authors (J.M. and J.D.) to validate the selection process shown in Table 1. This yielded an interrater agreement rate of 88% on article inclusion, which the authors deemed acceptable.

Remaining articles were screened for eligibility by title and abstract review by the same two reviewers (J.M. and J.D.) to remove articles that were not aligned with the inclusion/exclusion criteria (Table 1). Full texts were then screened for eligibility by both reviewers. Discrepancy between reviewers was resolved through discussion to achieve consensus.

### 2.4 Data Extraction

All data were extracted from Covidence and exported to Microsoft Excel 2024 by one author (J.M.), with a second author cross‐checking the exported data against the original paper (J.D.). Data included: bibliographic information, study funding, design (measures used), population information (sampling strategy, setting, data collection mode), outcomes of interest (prevalence of promotion associated with unhealthy foods and healthy foods, types of promotion), and statistical analyses (statistics used, significance of results). Article authors were contacted and asked to share additional data where the published results were not sufficient for synthesis.

### 2.5 Critical Appraisal

An adapted version of Hoy et al.'s (2012) checklist for assessing risk of bias in prevalence studies was used to analyze included studies [36]. External validity was assessed based on the study’s population sampling and sample selection. Internal validity was assessed across the source of data, case definition, reliable study instrument, mode of data collection, time of data collection, and appropriate parameters. This assessment was performed by a single author (J.M.), scoring each item as either low risk or high risk, with a second author (J.D.) cross‐checking the results. The risk of bias in the included studies was discussed in a qualitative manner, as recommended by Dekkers et al. (2019), given that the relevance of bias in observational studies is particularly context‐dependent [31].

### 2.6 Synthesis Methods

The key data extracted from each study were counts and proportion of total menu items having a promotional attribute and counts/proportion of unhealthy and healthy menu items within each promotional attribute. The odds ratios for healthy versus unhealthy menu items for each promotional attribute were also extracted if reported in the study [37, 38]. For studies where odds ratios had not been reported [39, 40, 41], analysis of the count data was undertaken to calculate the odds ratio for the likelihood of unhealthy menu items versus healthy menu items for each promotional attribute.

Two studies used a different approach to aggregating data, so excel data files containing the coded list of individual menu items were sourced from the authors [40, 41] and the counts and proportions were recalculated to match the subgroup analysis for this review. These data were summarized in tables according to where the data were sourced on the MDA (e.g., home page or outlet page) and by subgroups of promotional attributes (i.e., prioritized placement, images, combination deals, volume incentive, free delivery, discount, health claim, and other claim). Data from studies that investigated whole menus of outlets [39, 42, 43] (rather than menu items) were not tabulated and instead were described narratively. Given the heterogeneity of study methodologies, it was determined that a meta‐analysis was not possible. The certainty of synthesis findings was assessed through the number of studies, MDAs, menu items assessed, with the consistency of effects across studies and the risk of bias in studies.

### 2.7 Deviations From PROSPERO

Minor adjustments were made to this review methodology. Piloting the data extraction process was unnecessary as the relevant data were usually reported in a single table. A single reviewer extracted the data with a second reviewer cross‐checking all included studies. Longitudinal data were analyzed as a single measure rather than individual daily measures. Additionally, the Synthesis Without Meta‐analysis (SWiM) guidelines were used to guide reporting of data synthesis, as meta‐analysis was not possible (Table S3) [44].

## 3 Results

### 3.1 Search Results

**FIGURE 1:**
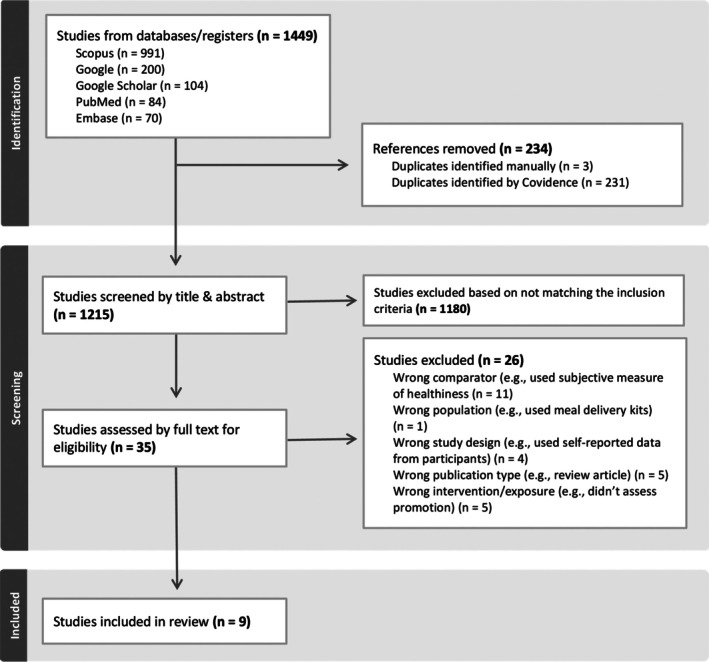
PRISMA flow chart illustrating study identification and screening results.

A total of 1449 articles were retrieved from the database searches. After title and abstract screening, 35 articles were reviewed in full‐text and nine articles were included in the review, see the PRISMA flowchart [32] in Figure 1.

### 3.2 General Study Characteristics

A summary of methods used in the nine included studies is provided in supplementary material (Table S4). Three studies were conducted in Brazil [40, 41, 43], two in New Zealand [37, 45], two in Australia [38, 46], and one in Canada [42], and one was a multicountry study conducted in Australia and New Zealand [39]. All studies employed a cross‐sectional design [37, 38, 39, 40, 42, 43, 45, 46], except for one with a longitudinal design [41]. Research in the field is recent, given the novelty of MDAs, with two studies having collected data in 2019 [42, 43], five in 2020 [37, 38, 39, 40, 41], and two in 2022 [45, 46]. All included studies were published in peer‐reviewed journals and no reports from gray literature were included.

**FIGURE 2:**
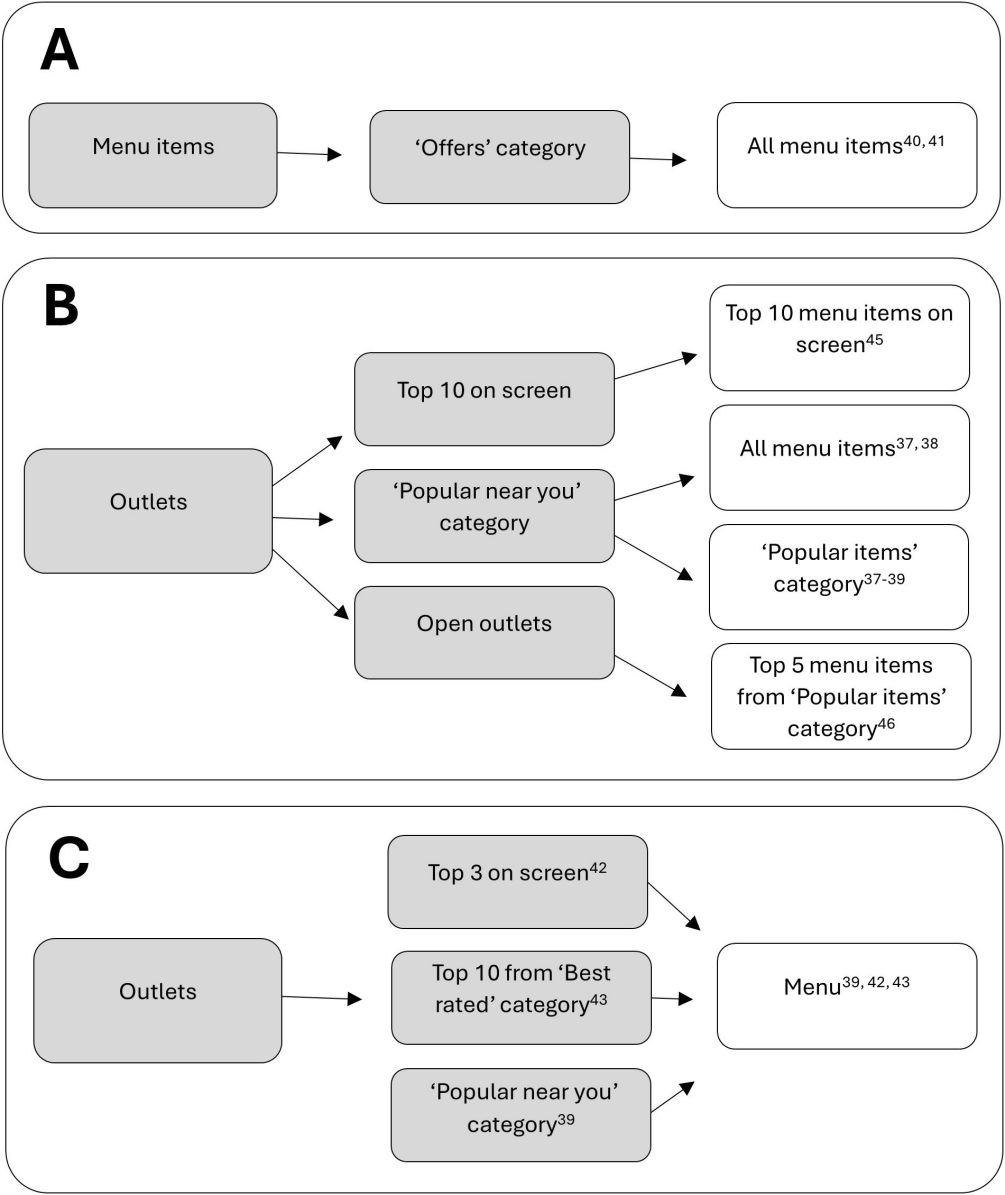
Results from studies assessing the following: (A) menus from meal delivery app home pages showing outlets; (B) menu items from meal delivery app home pages displaying menu items; and (C) menu items from meal delivery app home pages displaying outlets.

The nine studies could be categorized into three main groups, according to where the information was sourced on the MDA. Two studies used menu items from the home page [40, 41] (Figure 2A), and five studies used menu items from outlet pages [37, 38, 39, 45, 46] (Figure 2B). Three studies used whole menus of outlets [39, 42, 43] (Figure 2C)to collect data (note: This does not add up to nine, as one study collected information from two locations on the MDA interface and used different methods to assess their healthiness and promotional attributes [39]).

#### 3.2.1 Promotional Attributes

Promotional attributes assessed across the studies are defined in Table 2, with example images provided in Table S5. Four studies assessed the presence of images [37, 38, 40, 41]; three assessed combination deals [40, 41, 43], discounts [37, 40, 41] and volume incentives [37, 40, 41]; two assessed prioritized placement [37, 38], health claims [40, 41] and/or other claims [40, 41]; and one assessed free delivery offers [40].

**TABLE 2:** Promotional attributes on meal delivery apps, assessed in studies.

Promotional attribute		Definition	Promotional attributes assessed in included studies								
			**1** [42]	**2** [43]	**3** [40]	**4** [41]	**5** [39]	**6** [38]	**7** [37]	**8** [45]	**9** [46]
Prioritized placement	Outlet	Outlets with placement techniques that are likely to increase prominence. This includes appearing at the top of the screen, under a category such as “popular near you” or “best rated”	X	X	X	X	X	X	X	X	
	Item	Items with placement techniques that are likely to increase prominence. This includes appearing at the top of the screen or under a category such as “most popular items”					X	X	X	X	X
Image		Image accompanying items or outlets		X	X	X		X	X		
Combination deal (“Combo deal”)		Items bundled together (e.g., burger bundled with chips and drink), usually for a reduced cost compared to buying the items individually. Sometimes referred to as a “value bundle” or “meal deal”			X	X		X	X		
Price promotions	Discount	Any price reduction offered on an item or outlet spend		X	X	X					
	Free Delivery	An offer that removes delivery costs (e.g., “free delivery (spend $30)”)			X				X		
	Volume incentive	Any price promotion that gives greater volume to a purchase with proportionately reduced spending. This may include “buy‐1, get‐1‐free,” or “free item (spend $20)”			X	X			X		
Claims	Health	Any communication that indicates healthiness of an item or outlet. This includes being categorized as “healthy” or messages of healthiness in the menu or item description			X	X	X				
	Other	Any other communication that encourages purchasing a specific item or from an outlet by conveying its benefits			X	X					

### 3.3 Menu Items

The nine studies reported 29 outcomes associated with assessing the healthiness of menu items promoted on MDAs (Table 3). A total of 17 outcomes demonstrated greater odds of unhealthy menu items having a promotional attribute. Only three outcomes demonstrated greater odds of healthy items having a promotional attribute. Nine outcomes had no statistically significant difference between healthy and unhealthy menu items having a promotional attribute. Unhealthy items were more likely than healthy items to have prioritized menu placement and combination deals for all but one outcome in studies measuring these promotional attributes. Differences in outcomes were observed across studies for images and price promotions, with some showing increased odds for unhealthy items and others showing no difference. Notably, the only outcomes that found greater odds of healthy items having a promotional attribute were health claims. The differences in results may be due to different methodological approaches used across studies. [Correction added on 9 April 2026, after first online publication: The second, fourth, and fifth sentences in this paragraph have been updated in this version.]

**TABLE 3:** Summary of outcomes measuring the odds of unhealthy menu items compared to healthy menu items having a promotional attribute.

		↑ Unhealthy items	↑ Healthy items	No difference
Promotional attribute	Subcategory	*N*	*N*	*N*
Prioritized Items as a subsample of all menu items		2 [37, 38]		
Image				
		2 [37, 38]		
*Home page*				2 [40, 41]
*Prioritized menu items (outlet page)*		1 [37]		1 [38]
Combination deal				
*Home page*		2 [40, 41]		
*Outlet page*		2 [37, 38]		
*Prioritized menu items (outlet page)*		3 [38, 39]		1 [37]
Price promotions	Discount (*home page)*	1 [41]		1 [40]
	Volume Incentive *(home or outlet page)*	2 [40, 41]		1 [37]
	*Prioritized menu items (outlet page)*			1 [37]
	Free delivery (*home page)*			1 [40]
Claims	Health (*home page)*		2 [40, 41]	
	*Prioritized menu items (outlet page)*	1 [39]	1 [39]	
	Other (*home page)*	1 [40]		1 [41]

[Correction added on 9 April 2026, after first online publication: Some data in “Unhealthy items” and “No difference” columns has been corrected in this version.] *Note: N* indicates the number of statistically significant different outcomes in studies, with the results from Partridge et al. recorded for each geographic area separately rather than combined.

#### 3.3.1 Home Page Menu Items

As shown in Table 4, the healthiness of all menu items within the offers category of a home page was assessed in two studies [40, 41]. Both studies found that the majority of menu items were unhealthy, 65% for the first [41] and 71% for the second [40]. Images (88% [41] and 94% [40]) and discounts (92% [40] and 99% [41]) were found to be used often for both healthy and unhealthy items in both studies. Only one statistically significant result between the prevalence of discounts among healthy and unhealthy menu items was observed across the two studies [41], likely due to very low numbers of menu items not having a discount.

**TABLE 4:** Results of two studies assessing the healthiness of menu items with promotional attributes on the home page of meal delivery apps.

	Study	Total sample	Unhealthy, *n* (%)	Healthy, *n* (%)
**All menu items within** “**offers**” **category**	Horta, Matos & Mendes, 2021b [40]	1193	851 (71.3)	342 (28.7)
	Horta, Souza & Mendes, 2022 [41]	1115	726 (65.1)	389 (34.9)

Promotion		Prevalence*, n* (%)	*n* (% of unhealthy)	*n* (% of healthy)	OR (95% CI)
**Discount**	Horta, Matos & Mendes, 2021b [40]	1091 (91.5)	778 (91.4)	313 (91.5)	1.0 (0.6–1.5)
	Horta, Souza & Mendes, 2022 [41]	1104 (99.0)	723 (99.6)	381 (97.9)	5.1 (1.3–19.2)*
**Image**	Horta, Matos & Mendes, 2021b [40]	1125 (94.3)	802 (94.2)	323 (94.4)	1.0 (0.6–1.7)
	Horta, Souza & Mendes, 2022 [41]	982 (88.1)	635 (87.5)	347 (89.2)	0.8 (0.6–1.2)
**Free delivery**	Horta, Matos & Mendes, 2021b [40]	548 (45.9)	391 (45.9)	157 (45.9)	1.0 (0.8–1.3)
**Combination deal**	Horta, Matos & Mendes, 2021b [40]	437 (36.6)	405 (47.6)	32 (9.4)	8.8 (6.0–13.0)*
	Horta, Souza & Mendes, 2022 [41]	420 (37.7)	395 (54.4)	25 (6.4)	17.4 (11.3–26.7)*
**Volume incentive**	Horta, Matos & Mendes, 2021b [40]	203 (17.0)	181 (21.3)	22 (6.4)	3.9 (2.5–6.2)*
	Horta, Souza & Mendes, 2022 [41]	267 (23.9)	229 (31.5)	38 (9.7)	4.3 (2.9–6.2)*
**Other claim**	Horta, Matos & Mendes, 2021b [40]	145 (12.2)	116 (13.6)	29 (8.5)	1.7 (1.1–2.6)*
	Horta, Souza & Mendes, 2022 [41]	266 (23.8)	169 (23.3)	97 (24.9)	0.9 (0.7–1.2)
**Health claim**	Horta, Matos & Mendes, 2021b [40]	40 (3.4)	8 (0.9)	32 (9.4)	0.1 (0.0–0.2)*
	Horta, Souza & Mendes, 2022 [41]	46 (4.1)	0 (0.0)	46 (11.8)	N/A

*Note:* Odds ratios are reported as the odds of unhealthy menu items divided by the odds of healthy menu items having the promotional attribute.

*Indicates a statistically significant difference.

Around two fifths of menu items were a combination deal [40, 41]. Both studies found that unhealthy menu items were significantly more likely (OR: 8.8 (95% CI: 6.0–13.0) [40], OR: 17.4 (95% CI: 11.3–26.7) [41]) to be offered as a combination deal compared to healthy menu items. One study measured free delivery promotions and found that 45% of menu items had free delivery, with no differences between healthy and unhealthy menu items [40]. A smaller proportion of menu items had a volume incentive or health claim. Both studies found that unhealthy menu items were significantly more likely (OR: 3.9 (95% CI: 2.5–6.2) [40] and OR: 4.3 (95% CI: 2.9–6.2) [41]) to have a volume incentive compared to healthy menu items. Health claims were relatively uncommon overall, and unhealthy menu items were significantly less likely to have a health claim compared to healthy menu items (OR: 0.1 (95% CI: 0.0–0.02) [40]).

#### 3.3.2 Outlet Page Menu Items

As shown in Table 5, two studies assessed the healthiness and promotional attributes (images [37, 38], combination deals [37, 38], and volume incentives [37]) of all menu items within outlet pages of MDAs. In both studies, most menu items were unhealthy (73% [37] and 81% [38]). Substantial variation existed among the prevalence of menu items with images (30% [38] and 70% [37]) and those that were a value bundle (1.5% [37] and 8% [38]) across studies. Both studies found that unhealthy menu items were significantly more likely to have an image (OR: 1.3 (95% CI: 1.2–1.5) [38], OR: 1.7 (95% CI: 1.6–1.8) [37]) and to be part of a combination deal (OR: 4.6 (95% CI: 3.2–6.8) [37], OR: 6.5 (95% CI: 4.8–8.9) [38]) compared to healthy menu items.

**TABLE 5:** Results of studies assessing the healthiness of menu items with promotional attributes on the outlet pages of meal delivery apps.

	Study	Total sample	Unhealthy, *n* (%)	Healthy, *n* (%)
**All menu items**	Mahawar et al., 2022 [37]	25,877	18,955 (73.3)	6922 (26.7)
	Wang et al., 2021 [38]	13,841	11,139 (80.5)	2702 (19.5)

Promotion			Prevalence, *n* (% of total sample)	*n* (% of unhealthy)	*n* (% of healthy)	OR (95% CI)
**Image**	Mahawar et al., 2022 [37]		18,083 (69.9)	13,951 (73.6)	4132 (59.7)	1.7 (1.6–1.8)*
	Wang et al., 2021 [38]		4097 (29.6)	3420 (30.7)	677 (25.1)	1.3 (1.2–1.5)*
**Combination deal** ^**a**^	Mahawar et al., 2022 [37]		392 (1.5)	363 (1.9)	29 (0.4)	4.6 (3.2–6.8)*
	Wang et al., 2021 [38]		1107 (8.0)	1064 (9.6)	43 (1.6)	6.5 (4.8–8.9)*
**Volume incentive** ^**b**^	Mahawar et al., 2022 [37]		356 (1.4)	265 (1.4)	91 (1.3)	1.1 (0.8–1.4)

Prioritized placement			Total sample	Unhealthy *n* (%)	Healthy *n* (%)
**Sample included only prioritized menu items**	Partridge et al., 2020 [39]	Auckland	2412	2128 (88.2)	284 (11.8)
		Sydney	3357	2830 (84.3)	527 (15.7)
		Total	5769	4958 (85.9)	811 (14.1)
	Norriss et al., 2022 [45]	Menulog	8076	6558 (81.2)	1518 (18.8)
		UberEats	7458	5752 (77.1)	1706 (22.9)
		DeliverEasy	5337	3887 (72.8)	1450 (27.2)
		Total	20,871	16,197 (77.6)	4674 (22.4)
	Cassano et al., 2024 [46]		452	345 (76.3)	107 (23.7)

			Prevalence*, n* (%of total sample)	*n* (% of unhealthy)	*n* (% of healthy)	OR (95% CI)
**Prioritized menu items as a subsample of all menu items**	Mahawar et al., 2022 [37]		1571 (6.1)	1317 (6.9)	254 (3.7)	2.0 (1.7–2.2)*
	Wang et al., 2021 [38]		625 (4.5)	568 (5.1)	57 (2.1)	2.5 (1.9–3.2)*

Promotion						
**Image**	Mahawar et al., 2022 [37]		1234 (78.5)	1089 (82.7)	145 (57.1)	3.6 (2.7–4.8)*
	Wang et al., 2021 [38]		551 (88.2)	501 (88.2)	50 (87.7)	1.0 (0.5–2.4)
**Combination deal** ^**a**^	Mahawar et al., 2022 [37]		53 (3.4)	47 (3.6)	6 (2.4)	1.5 (0.6–3.6)
	Wang et al., 2021 [38]		53 (8.5)	53 (9.3)	0	N/A
	Partridge et al., 2020 [39]	Auckland	700 (29.0)	700 (32.0)	0	N/A
		Sydney	289 (8.6)	289 (10.2)	0	N/A
		Total	989 (17.1)	989 (19.9)	0	N/A
**Health claim**	Partridge et al., 2020 [39]	Auckland	330 (13.7)	243 (11.4)	87 (30.6)	0.3 (0.2–0.4)*
		Sydney	532 (15.8)	469 (16.6)	63 (12.0)	1.5 (1.1–1.9)*
		Total	862 (14.9)	712 (14.4)	150 (18.5)	0.7 (0.6–0.9)*
**Volume incentive** ^**b**^	Mahawar et al., 2022 [37]		15 (1.0)	14 (1.1)	1 (0.4)	2.7 (0.4–20.8)

*Note:* Odds ratios are reported as the odds of unhealthy menu items divided by the odds of healthy menu items having the promotional attribute.

*Indicates a statistically significant difference.

^a^Set meals (e.g., fish and chips) were excluded from Mahawar et al.'s analysis of combination deals.

^b^Free delivery offers included in volume incentives.

Among the five studies that included prioritized menu items, all found that the majority were unhealthy (77% [46] to 90% [38]) (Table 5). Two of the three studies that assessed combination deals among prioritized menu items found that they were only offered for unhealthy menu items [38, 39], while the other study found that prioritized unhealthy menu items were more likely to be a combination deal compared to prioritized healthy menu items [37]. One study assessed the prevalence of healthy and unhealthy menu items within prioritized outlet menus that displayed a health claim [39]. Overall, 15% (*n* = 862) of menu items were from outlet menus with a health claim, of which 17% (*n* = 150) of menu items were classified as healthy [39]. Of note, the presence of unhealthy menu items, compared to healthy items, within outlet menus with a health claim was more likely in Sydney (OR: 1.5 [95% CI: 1.1–1.9]) and less likely in Auckland (OR: 0.3 [95% CI: 0.2–0.4]) [39].

Two studies compared the proportion of prioritized menu items to regular menu items and found that 4.5% [38] and 6.1% [37] of all menu items were prioritized, respectively (Table 5). In both studies, unhealthy menu items were more likely (OR: 2.5, 95% CI 1.9–3.2) [38] and (OR: 2.0, 95% CI 1.7–2.2) [37] to be prioritized compared to healthy menu items.

### 3.4 Outlet Menus

The healthiness of menus from prioritized outlets (higher on the screen of MDAs) was assessed in three studies, each using a different measure to classify menus [39, 42, 43]. The results across all three studies showed that most outlet menus were unhealthy. The 12 menus that Brar and Minaker assessed all scored between 20 and 51 out of 100 on the *Healthy Eating Index‐2015*, which does not include criteria for distinguishing healthy from unhealthy scores [42]. However, using the criteria from *Healthy Eating Index‐2000*, which states that scores between 51–80 “need improvement” and scores below 50 are “poor,” all of the scores from Brar and Minaker's analysis are indicative of unhealthy menus [47]. Horta et al. (2021a) assessed 362 outlet menus and found that the majority offered unhealthy items; for example, 78% of these menus had ultra‐processed beverages while only 49% had water [43]. Similarly, Partridge et al. (2020) [39] assessed 1074 outlet menus and found that 73% of these were unhealthy according to the *Food Environment Score* (−10 to −5), 22% were less healthy (−4 to 4), and 5% were healthy (5 to 10).

### 3.5 Study Methodology

High methodological variation existed across studies, in part due to the MDAs included across the included studies having different configurations for displaying outlets and menu items. For example, the home page of some MDAs displayed both outlets and individual menu items whereas other MDAs only displayed menu items within the outlet page. Variation across studies also existed in their scope (e.g., all menu items within an outlet, vs. prioritized menu items only) and location (e.g., home page or outlet page) of the menu item data that were collected (Table S4).

The number of outlets or menu items extracted and the number of geographical areas covered also differed substantially between studies (Table S4). Five studies included one MDA in their analysis [37, 38, 39, 40, 41] and the remaining studies included between two and four MDAs [42, 43, 45, 46]. UberEats was assessed in six studies [37, 38, 39, 42, 45, 46], Menulog in two studies [45, 46], and DeliverEasy [45], Deliveroo [46], DoorDash [42], Foodora [42], and Skipthedishes [42] each assessed in one study. The names of the MDAs used in the three studies conducted in Brazil [40, 41, 43] were not disclosed. The healthiness of foods was assessed using a variety of measures across the studies (Table S4). Guidelines that used an unhealthy/healthy dichotomy were employed in eight studies, albeit with slightly different definitions; four used the *Australian Dietary Guidelines* [37, 38, 39, 46], three used the *NOVA food classification system* endorsed by the *Brazilian Dietary Guidelines* [40, 41, 43], and one used the *Eating and Activity Guidelines for New Zealand Adults* [45]. Another two studies utilized classification systems that scored menu components on a scale, including the *Healthy Eating Index‐2015* [42] and the *Food Environment Score* [39].

#### 3.5.1 Risk of Bias

All studies were assessed as having an overall low risk of bias and further research is unlikely to change our confidence in the findings (Item 10, Table 6). Six studies had a low risk of bias on nine out of 10 items [37, 39, 41, 43, 45, 46], and three studies had a low risk of bias on eight out of 10 items [38, 40, 42]. All studies used appropriate sample populations and study definitions and instruments, and most studies used appropriate data collection procedures. The most common factor contributing to a higher risk of bias in eight out of nine studies was that nutritional status of the food items was coded based on averages for a category of food rather than the nutritional information being collected from the MDA or outlet website (e.g., from ingredient list or kilojoule labelling). This may not reflect the true range of healthiness a food can be (i.e., two burgers could have very different nutrient profiles). The overall risk of bias in the studies is unlikely to change the findings. However, the way in which MDAs promote foods is likely to change over time and thus the healthiness of foods promoted on MDAs is likely to change, not as a result of study methodology but rather the perpetual evolvement of such digital technologies.

**TABLE 6:** Results from risk of bias assessments.

Risk of bias item		*n* (studies with low risk)	%
1.	Was the study's target population a close representation to the population in relation to relevant variables? *(*i.e.*, investigated meal delivery apps in a highly populated city)*	9	100
2.	Was the sampling frame a true or close representation of the target population? *(*i.e.*, assessed foods from independent and chain outlets)*	7	78
3.	Was some form of random selection used to select the sample, OR, was a census undertaken? *(*i.e.*, all items or outlets assessed, or a random sample)*	9	100
4.	Were data collected directly from the meal delivery apps? *(*i.e.*, nutritional data collected as displayed on the meal delivery apps)*	1	11
5.	Was an acceptable case definition used in the study? *(*i.e.*, meal delivery app and included foods were defined)*	9	100
6.	Was the study instrument that measured the prevalence of promotions and the nutritional quality of foods shown to have reliability and validity? *(*i.e.*, formal guidelines were used to assess healthiness)*	9	100
7.	Was the same mode of data collection used for all subjects? *(*i.e.*, all data were extracted automatically)*	8	89
8.	Was the time of data collection appropriate? *(*i.e.*, data were collected at common mealtimes)*	8	89
9.	Were the numerator(s) and denominator(s) for the parameters appropriate? *(*i.e.*, the prevalence of promotions was assessed in comparison to the number of menu items)*	9	100
10.	Summary item on the overall risk of study bias *(*i.e.*, further research is unlikely to change our confidence in the estimate)*	9	100

## 4 Discussion

This review systematically synthesized data on the healthiness of foods promoted on MDAs. The included studies spanned four countries (Australia, New Zealand, Canada, and Brazil) and were highly heterogeneous in methodological approach, which meant that there was a range of MDAs evaluated (i.e., UberEats, Menulog, DeliverEasy, Deliveroo, DoorDash, Foodora, Skipthedishes, and undisclosed MDAs from Brazil), each presenting varied promotional strategies. A meta‐analysis was not possible, but findings from this review do indicate that unhealthy menu items on MDAs are more likely than healthy items to have certain promotional attributes, including having prioritized placement, being part of a combination deal, and having a volume incentive.

Prioritized placement of unhealthy menu items shown in this review increases awareness and salience of unhealthy foods, which likely encourages the purchase of unhealthy over healthy foods [21]. The UK government has enforced restrictions on the display of unhealthy foods in prominent locations of grocery retail stores, including the “picked for you” section of online grocery home pages (*The Food (Promotion and Placement) (England) Regulations 2021*, enacted 2022) [48, 49]. Public health experts recommend similar policies be applied to MDAs to promote the purchase of healthy menu items [27]. An experimental study in the UK found that repositioning healthy outlets on the home page and healthy menu items on the outlet page of a simulated MDA significantly reduced calories (−209 kcal) in participants’ food choices compared to the control condition (1382 kcal) [50]. This provides promising evidence that placement policies in MDAs could improve the healthiness of food choices.

Outlets on MDAs can self‐classify into certain food categories such as “healthy,” which is a more subtle promotional strategy. This serves as a blanket health claim, applied to the entire menu, rather than individual menu items [51]. Blanket health claims were uncommon in the two studies from Brazil included in this review [40, 41], but one study conducted in Australia and New Zealand did find that around 15% of menu items were from outlets that were self‐categorized as healthy. This is contrary to previous research that investigated the prevalence of health claims on 44 Australian fast food websites and found no blanket health claims [51]. Hence, the novel format of MDAs may serve as an opportunity for outlets to use blanket health claims as a promotional strategy. Concerningly, one study from this review found that, among the sample from Sydney, unhealthy menu items were more likely than healthy items to be in outlets with blanket health claims. Evidence from studies examining consumer choices in various simulated retail food environments has demonstrated that the presence of health claims on foods is associated with greater perceived product healthiness and increased product purchasing [52, 53]. Given the impact of health claims in the retail food environment, the display of such claims on MDAs and their impact on consumer choice should be further explored.

Another common promotional strategy used within online and offline food environments is value pricing. Previous research assessing the price of menu items in fast food outlets has shown a value‐pricing effect, whereby higher‐calorie menu items were less expensive per calorie than lower‐calorie menu items [54, 55]. This aligns with findings from the present review where value pricing, including volume incentives, was found to be more common for unhealthy menu items. Furthermore, it is well established that price promotions increase food purchasing, particularly for unhealthy foods [21, 56]. Qualitative research investigating perceptions of UK adults who frequently used MDAs found that price promotions were commonly cited as a reason for justifying use [57]. While the effect of price promotions on consumer food choice on MDAs has yet to be comprehensively explored, given the established impact of price promotions on purchasing behavior in other food environments, public health experts have suggested that policies limiting price promotions for unhealthy menu items and increasing those for healthy menu items on MDAs are likely to improve dietary choices [27]. Similar policies have been implemented in other food environments. For example, the UK government has implemented a policy restricting volume incentives in grocery retail (*The Food (Promotion and Placement) (England) Regulations 2021*, enacted 2022) [48, 49]. This policy could be translated to MDAs for real‐world testing and evaluation.

In the same domain as value pricing, combination deals are commonly employed by fast food companies. This review found that unhealthy menu items were more likely to be offered as a combination deal compared to healthy menu items. This aligns with research from Australia investigating the healthiness of items from fast food outlets [58]. Howes and colleagues found that the average meal deal included default sides of fries and soft drinks and contributed to over half of the recommended daily energy intake for adults [58]. Policies that mandate the default sides to be healthier options (e.g., replacing fries with a salad) could be used to encourage healthier food choices [27]. Furthermore, a study from the USA found that higher‐calorie combination deals (e.g., upsized to large and addition of condiments offered) provided significantly more calories per dollar spent [59]. The studies included in this review did not assess add‐ons (e.g., extra sauce or side of chips) that appear on MDAs [60, 61, 62]. Financial incentives that encourage larger food orders likely contribute to increased energy intake, presenting a further opportunity for intervention.

### 4.1 Strengths and Limitations

This review had many strengths, including a comprehensive and systematic search strategy, and broad inclusion criteria to capture the diversity of approaches taken to document promotions used on MDAs. The authors were contacted to provide clarity or additional data where the published results were insufficient to synthesize. Our approach resulted in a heterogeneous group of studies that could not be synthesized using meta‐analysis. A narrative approach to data synthesis was used to provide an indicator of the current state of evidence, imperative to guide future research in this rapidly evolving environment.

The current evidence on promotional strategies used by MDAs is limited by numerous methodological issues. For example, the included studies were mostly cross‐sectional in design, which does not account for the highly dynamic nature of promotional attributes on MDAs. Included studies were conducted in a small number of countries, with no relevant studies done in countries with the highest use of MDAs, including China and the USA [63], thus limiting the generalizability of findings. The studies used different sampling strategies (e.g., extracting menu items from the outlet page vs. the home page) and had different definitions of promotional attributes, particularly regarding prioritized placement (e.g., top 10 items on screen versus “popular near you” category). Another example was “free delivery,” which was included in volume incentives by one study [37] and measured as an independent outcome in another study [40]. Similarly, Wang et al. [38] included combination meals (e.g., fish and chips) in combination deals, while Mahawar et al. [37] did not. Additionally, different measures were used to assess the healthiness of menu items across studies. The *NOVA food classification system*, which classifies food according to their degree of processing, was used by some studies [40, 41, 43]. While the *Australian Dietary Guidelines*, which classifies food according to whether they provide necessary nutrients to the body, was used by other studies [37, 38, 39, 45]. Longitudinal designs and consistent sampling frames and definitions are needed to improve monitoring of the healthiness of menu items and promotional attributes on MDAs.

## 5 Conclusion

MDAs increase accessibility to unhealthy food, which likely increases consumption of these foods. Understanding the environment in which people make food choices is vital to improving population health outcomes. This review found that unhealthy foods on MDAs are more likely than healthy foods to have promotional attributes, including prioritized placement and combination deals, encouraging the purchase of unhealthy foods. Methodological improvements, including standardized terminology and definitions regarding promotional attributes on MDAs, are needed to facilitate the integration of findings across MDA platforms and countries. Quantifying the full extent of promotional attributes used by MDAs, not just in subsections such as outlet pages, will be important for demonstrating the need for regulation. Experimental testing of the effects of promotional attributes on consumer choice in an MDA environment is also needed. Together, this evidence can inform the development of appropriate policy interventions that aim to improve this currently unregulated food environment to better support healthy food choices.

## Author Contributions

All authors contributed to the work's conception and design. J.M. performed the literature search and wrote the manuscript. J.M. and J.D. performed the screening, identified eligible studies. J.D. validated data extraction and risk of bias assessments. C.M., J.D., and S.P. assisted in manuscript writing.

## Funding

This work was done as part of the “Healthy Food, Healthy Planet, Healthy Planet” Centres of Research Excellence funded by the National Health and Medical Research Council (NHMRC) grant (2006620). J.M. is supported by The University of Adelaide Research Scholarship. J.D. is a Beat Cancer Early Career Research Fellow, receiving *financial and other support of Cancer Council SA's Beat Cancer Project on behalf of its donors and the State Government of South Australia through the Department of Health and Wellbeing*. CM is supported by NHMRC Investigator grant (GNT 1195421). Sources of financial support had no involvement in this research.

## Conflicts of Interest

The authors declare no conflicts of interest.

## Supporting information


**Table S1:** PRISMA checklist.
**Table S2:** Search strategy used in PubMed.
**Table S3:** Synthesizes without meta‐analysis checklist.
**Table S4:** Summary of included study methods and sample characteristics.
**Table S5:** Example images of promotional strategies used on meal delivery apps.

## Data Availability

Data sharing not applicable to this article as no datasets were generated or analysed during the current study.
