# Supplementary material for: Combo Deals, Junk Meals: A Systematic Review Examining the Healthiness of Foods Promoted on Meal Delivery Apps
Source: Obes Rev. 2026 Feb 9;27(7):e70097. doi: 10.1111/obr.70097 (PMC13243348; doi:10.1111/obr.70097)
Supplement: Supplementary file 1 — Table S1: PRISMA checklist. Table S2: Search strategy used in PubMed. Table S3: Synthesizes without meta‐analysis checklist. Table S4: Summary of included study methods and sample characteristics. Table S5: Example images of promotional strategies used on meal delivery apps. [file OBR-27-e70097-s001.docx]

**Table S1:** PRISMA checklist

| **Section and Topic** | **Item #** | **Checklist item** | **Location where item is reported** |
| --- | --- | --- | --- |
| **TITLE** | | |  |
| Title | 1 | Identify the report as a systematic review. | Page 1 |
| **ABSTRACT** | | |  |
| Abstract | 2 | See the PRISMA 2020 for Abstracts checklist. | Abstract, Page 1 |
| **INTRODUCTION** | | |  |
| Rationale | 3 | Describe the rationale for the review in the context of existing knowledge. | Introduction, Pages 1 & 2 |
| Objectives | 4 | Provide an explicit statement of the objective(s) or question(s) the review addresses. | Introduction, Pages 1 & 2 |
| **METHODS** | | |  |
| Eligibility criteria | 5 | Specify the inclusion and exclusion criteria for the review and how studies were grouped for the syntheses. | Methods, Page 2 |
| Information sources | 6 | Specify all databases, registers, websites, organisations, reference lists and other sources searched or consulted to identify studies. Specify the date when each source was last searched or consulted. | Methods, Page 2 |
| Search strategy | 7 | Present the full search strategies for all databases, registers and websites, including any filters and limits used. | Methods, Pages 2 & 3, Supplementary Table S2 |
| Selection process | 8 | Specify the methods used to decide whether a study met the inclusion criteria of the review, including how many reviewers screened each record and each report retrieved, whether they worked independently, and if applicable, details of automation tools used in the process. | Methods, Page 3 |
| Data collection process | 9 | Specify the methods used to collect data from reports, including how many reviewers collected data from each report, whether they worked independently, any processes for obtaining or confirming data from study investigators, and if applicable, details of automation tools used in the process. | Methods, Pages 9 & 10 |
| Data items | 10a | List and define all outcomes for which data were sought. Specify whether all results that were compatible with each outcome domain in each study were sought (e.g. for all measures, time points, analyses), and if not, the methods used to decide which results to collect. | Methods, Page 3 |
|  | 10b | List and define all other variables for which data were sought (e.g. participant and intervention characteristics, funding sources). Describe any assumptions made about any missing or unclear information. | Methods, Page 3 |
| Study risk of bias assessment | 11 | Specify the methods used to assess risk of bias in the included studies, including details of the tool(s) used, how many reviewers assessed each study and whether they worked independently, and if applicable, details of automation tools used in the process. | Methods, Page 3 |
| Effect measures | 12 | Specify for each outcome the effect measure(s) (e.g. risk ratio, mean difference) used in the synthesis or presentation of results. | N/A |
| Synthesis methods | 13a | Describe the processes used to decide which studies were eligible for each synthesis (e.g. tabulating the study intervention characteristics and comparing against the planned groups for each synthesis (item #5)). | Methods, Pages 3 & 4 |
|  | 13b | Describe any methods required to prepare the data for presentation or synthesis, such as handling of missing summary statistics, or data conversions. | Methods, Pages 3 & 4 |
|  | 13c | Describe any methods used to tabulate or visually display results of individual studies and syntheses. | Methods, Pages 3 & 4 |
|  | 13d | Describe any methods used to synthesize results and provide a rationale for the choice(s). If meta-analysis was performed, describe the model(s), method(s) to identify the presence and extent of statistical heterogeneity, and software package(s) used. | Methods, Pages 3 & 4 |
|  | 13e | Describe any methods used to explore possible causes of heterogeneity among study results (e.g. subgroup analysis, meta-regression). | N/A |
|  | 13f | Describe any sensitivity analyses conducted to assess robustness of the synthesized results. | N/A |
| Reporting bias assessment | 14 | Describe any methods used to assess risk of bias due to missing results in a synthesis (arising from reporting biases). | N/A |
| Certainty assessment | 15 | Describe any methods used to assess certainty (or confidence) in the body of evidence for an outcome. | N/A |
| **RESULTS** | | |  |
| Study selection | 16a | Describe the results of the search and selection process, from the number of records identified in the search to the number of studies included in the review, ideally using a flow diagram. | Results, Page 4, Figure 1 |
|  | 16b | Cite studies that might appear to meet the inclusion criteria, but which were excluded, and explain why they were excluded. | Results, Page 4, Figure 1 |
| Study characteristics | 17 | Cite each included study and present its characteristics. | Results, Pages 4 & 5, Supplementary Table S3 |
| Risk of bias in studies | 18 | Present assessments of risk of bias for each included study. | Results, Pages 10 & 11, Table 6 |
| Results of individual studies | 19 | For all outcomes, present, for each study: (a) summary statistics for each group (where appropriate) and (b) an effect estimate and its precision (e.g. confidence/credible interval), ideally using structured tables or plots. | Results, Tables 3-5 |
| Results of syntheses | 20a | For each synthesis, briefly summarise the characteristics and risk of bias among contributing studies. | N/A |
|  | 20b | Present results of all statistical syntheses conducted. If meta-analysis was done, present for each the summary estimate and its precision (e.g. confidence/credible interval) and measures of statistical heterogeneity. If comparing groups, describe the direction of the effect. | N/A |
|  | 20c | Present results of all investigations of possible causes of heterogeneity among study results. | N/A |
|  | 20d | Present results of all sensitivity analyses conducted to assess the robustness of the synthesized results. | N/A |
| Reporting biases | 21 | Present assessments of risk of bias due to missing results (arising from reporting biases) for each synthesis assessed. | N/A |
| Certainty of evidence | 22 | Present assessments of certainty (or confidence) in the body of evidence for each outcome assessed. | N/A |
| **DISCUSSION** | | |  |
| Discussion | 23a | Provide a general interpretation of the results in the context of other evidence. | Discussion, Pages 11 & 12 |
|  | 23b | Discuss any limitations of the evidence included in the review. | Discussion, Page 12 |
|  | 23c | Discuss any limitations of the review processes used. | Discussion, Page 12 |
|  | 23d | Discuss implications of the results for practice, policy, and future research. | Discussion, Pages 11 & 12 |
| **OTHER INFORMATION** | | |  |
| Registration and protocol | 24a | Provide registration information for the review, including register name and registration number, or state that the review was not registered. | Methods, Page 2 |
|  | 24b | Indicate where the review protocol can be accessed, or state that a protocol was not prepared. | Methods, Page 2 |
|  | 24c | Describe and explain any amendments to information provided at registration or in the protocol. | Methods, Page 4 |
| Support | 25 | Describe sources of financial or non-financial support for the review, and the role of the funders or sponsors in the review. | Acknowledgements, Page 13 |
| Competing interests | 26 | Declare any competing interests of review authors. | Page 13 |
| Availability of data, code and other materials | 27 | Report which of the following are publicly available and where they can be found: template data collection forms; data extracted from included studies; data used for all analyses; analytic code; any other materials used in the review. | Supplementary File 1 |

*From:*  Page MJ, McKenzie JE, Bossuyt PM, Boutron I, Hoffmann TC, Mulrow CD, et al. The PRISMA 2020 statement: an updated guideline for reporting systematic reviews. BMJ 2021;372:n71. doi: 10.1136/bmj.n71. This work is licensed under CC BY 4.0. To view a copy of this license, visit <https://creativecommons.org/licenses/by/4.0/>

**Table S2:** Search strategy used in PubMed

| **Meal delivery app** | **Promotion or health** |
| --- | --- |
| On-demand food delivery[tiab] OR Food delivery app[tiab] OR Meal delivery app[tiab] OR Mobile food delivery[tiab] OR App-based order*[tiab] OR Online food delivery[tiab] OR Food delivery service[tiab] | health* OR promo* OR market* |

**Table S3:** Synthesises Without Meta-analysis checklist

| **SWiM reporting item** | **Item description** | **Page in manuscript where item is reported** |
| --- | --- | --- |
| *Methods* | | |
| **1** Grouping studies for synthesis | 1a) Provide a description of, and rationale for, the groups used in the synthesis (e.g., groupings of populations, interventions, outcomes, study design) | 13 & 14 |
|  | 1b) Detail and provide rationale for any changes made subsequent to the protocol in the groups used in the synthesis | N/A |
| **2** Describe the standardised metric and transformation methods used | Describe the standardised metric for each outcome. Explain why the metric(s) was chosen, and describe any methods used to transform the intervention effects, as reported in the study, to the standardised metric, citing any methodological guidance consulted | 12 |
| **3** Describe the synthesis methods | Describe and justify the methods used to synthesise the effects for each outcome when it was not possible to undertake a meta-analysis of effect estimates | 12 |
| **4** Criteria used to prioritise results for summary and synthesis | Where applicable, provide the criteria used, with supporting justification, to select the particular studies, or a particular study, for the main synthesis or to draw conclusions from the synthesis (e.g., based on study design, risk of bias assessments, directness in relation to the review question) | 12 |
| **5** Investigation of heterogeneity in reported effects | State the method(s) used to examine heterogeneity in reported effects when it was not possible to undertake a meta-analysis of effect estimates and its extensions to investigate heterogeneity | 12 |
| **6** Certainty of evidence | Describe the methods used to assess certainty of the synthesis findings | 12, 13 |
| **7** Data presentation methods | Describe the graphical and tabular methods used to present the effects (e.g., tables, forest plots, harvest plots).  Specify key study characteristics (e.g., study design, risk of bias) used to order the studies, in the text and any tables or graphs, clearly referencing the studies included | 12 |
| *Results* | | |
| **8** Reporting results | For each comparison and outcome, provide a description of the synthesised findings, and the certainty of the findings. Describe the result in language that is consistent with the question the synthesis addresses, and indicate which studies contribute to the synthesis | 17-22 |
| *Discussion* | | |
| **9** Limitations of the synthesis | Report the limitations of the synthesis methods used and/or the groupings used in the synthesis, and how these affect the conclusions that can be drawn in relation to the original review question | 23-27 |

PRISMA=Preferred Reporting Items for Systematic Reviews and Meta-Analyses.

*If the information is not provided in the systematic review, give details of where this information is available (e.g., protocol, other published papers (provide citation details), or website (provide the URL)).

*From:* The citation for the Synthesis Without Meta-analysis explanation and elaboration article is: Campbell M, McKenzie JE, Sowden A, Katikireddi SV, Brennan SE, Ellis S, Hartmann-Boyce J, Ryan R, Shepperd S, Thomas J, Welch V, Thomson H. Synthesis without meta-analysis (SWiM) in systematic reviews: reporting guideline BMJ 2020;368:l6890 <http://dx.doi.org/10.1136/bmj.l6890>

**Table S4:** Summary of included study methods and sample characteristics

| Study | Design, country, data collection year | Sample selection | Sample Size (*n*) | | | Promotion assessed | Measure of healthiness |
| --- | --- | --- | --- | --- | --- | --- | --- |
|  |  |  | **Meal delivery apps** | **Outlets** | **Menu items** |  |  |
| Brar & Minaker, 2021^42^ | Cross-sectional  Canada  2019 | Searches were done in 1 postal code in a major city.  Assessed the full menu of the first 3 outlets on meal delivery app (UberEats, DoorDash, SkiptheDishes and Foodora) home pages. | 4 | 12 | 759 | Prioritised outlets | Dietary constituents of the outlets menu (based on website description or The United States Department of Agriculture Food and Nutrient Database of Dietary Studies) were summed together and scores of each of the 13 components (e.g., total vegetables, added sugars) in the Healthy Eating Index – 2015 were assigned producing an overall menu score (maximum 100 points), relative to alignment with the 2015-2020 Dietary Guidelines for Americans. |
| Horta et al., 2021a^43^ | Cross-sectional  Brazil  2019 | Searches were done in 18 randomly selected neighbourhoods (two from each of the administrative regions) in a major city.  Assessed full menus of the first 10 outlets under the best-rated category of meal delivery app (undisclosed names) home pages were included in analysis. | 2 | 362 | N/A | Prioritised outlets, images, discounts | Menu items were classified into 10 food and beverage groups according to whether they contained ultra-processed foods using the NOVA food classification system and Brazilian Dietary Guidelines. Predominantly healthy food and beverage groups included: water and traditional meals. Predominantly ultra-processed food and beverage groups included ultra-processed beverages and fried savoury snacks. |
| Horta, Matos & Mendes, 2021b^40^ | Cross-sectional  Brazil  2020 | A search was done in the capital city of each Brazilian state (*n*=27).  Assessed a random 25% sample of items under the ‘offers’ category on a meal delivery app (undisclosed name) home page*.* | 1 | N/A | 1,754 | Prioritised outlets, images free delivery, images, discounts, value bundles, volume incentives, health claims, other claims |  |
| Horta Souza & Mendez, 2022^41^ | Longitudinal  Brazil  2020 | Searches were done in the capital city of a major Brazilian city (*n*=1).  Assessed a random 25% sample of items under the ‘offers’ category on a meal delivery app (undisclosed name) home page*.* | 1 | N/A | 1,593 | Prioritised outlets, images, discounts, value bundles, volume incentives, health claims, other claims |  |
| Partridge et al., 2020^39^ | Cross-sectional  Australia and New Zealand  2020 | Searches were done in areas with a higher-than-average concentration (>30%) of young people (15-34, primary meal delivery app users) in major cities.  Syndey *N*=233 suburbs searched  Auckland *N*= 186 suburbs searched  Assessed menus of top 10 outlets under ‘popular near you’ category and ‘most popular’ menu items in each outlet on the meal delivery app (UberEats). | 1 | 1,074 | 5,769 | Prioritised outlets, prioritised menu items, health claims | Menu items were classified, based on their description, as core (e.g., vegetables, grains) or discretionary (food and beverages high in unhealthy nutrients, including saturated fats), according to the Australian Dietary Guidelines. The Australian Bureau of Statistics discretionary food list was used for assistance.  Outlet menus were assessed using an adapted version of the Food Environment Score (FES), which classified outlets into 18 categories and scored according to their ‘healthiness’ into three groups: healthy (FES range +5 to +10); less healthy (FES range −4 to +4) and unhealthy (FES range −10 to −5). |
| Wang et al., 2021^38^ | Cross-sectional  Australia  2020 | Search method see Partridge et al., 2020 ^5^ for Sydney.  Assessed all menu items in top 10 outlets under ‘popular near you’ category. Only includes independent outlets (excludes chains). | 1 | 196 | 13,841 | Prioritised outlets, prioritised menu items, images, value bundles | Menu items were coded into 38 food and beverage categories. Australian Dietary Guidelines were then used to categorise these by Five Food Group (FFG) (e.g., vegetables, grains) or discretionary (foods high in unhealthy nutrients, including saturated fats). The Australian Bureau of Statistics principles and list for identifying discretionary foods was used to assist coding. |
| Mahawar et al., 2022^37^ | Cross-sectional  New Zealand  2020 | Search method see Partridge et al., 2020 ^5^ for Auckland.  Assessed all menu items in top 10 outlets under ‘popular near you’ category. | 1 | 354 | 29,764 | Prioritised outlets, prioritised menu items, images, value bundles**,** volume incentives**,** free delivery |  |
| Norriss et al., 2022^45^ | Cross-sectional  New Zealand  2022 | 81 representative addresses chosen from a major city.  Assessed top ten menu items on outlet page of top 10 outlets on the home pages of meal delivery apps (UberEats, Menulog, Delivereasy). | 3 | 2,200 | 20,871 | Prioritised outlets, prioritised menu items | Menu items were coded into 36 food and beverage categories which were assigned a based on core components (e.g., ‘variety’, ‘fruit/veg’) and discretionary components (e.g., ‘saturated fat’, ‘processed’), with reference to the Eating and Activity Guidelines for New Zealand Adults. This gave menu item components a score as either healthy (1 or 2) or unhealthy (-2, -1, or 0). A machine-learning model, was used to code the sample. |
| Cassano et al., 2024^46^ | Cross-sectional  Australia  2022 | The top 10 areas with the highest population of young people (15-44, primary meal delivery app users), with a representative spread of socioeconomic disadvantage, giving 10 addresses.  Assessed top 5 menu items in the ‘popular items’ section in outlets on Menulog and Deliveroo and in the ‘picked for you’ category on UberEats. | 3 | 92 | 452 | Prioritised menu items | Menu items were coded into 48 food and beverage categories, which aligned to the Australian Dietary Guidelines classification of food as either Five Food Group (FFG) (e.g., vegetables, grains) or discretionary (foods high in unhealthy nutrients, including saturated fats). |

**Table S5:** Example images of promotional strategies used on meal delivery apps.

| **Promotional Attribute** | **Example image** |
| --- | --- |
| Prioritised outlet | 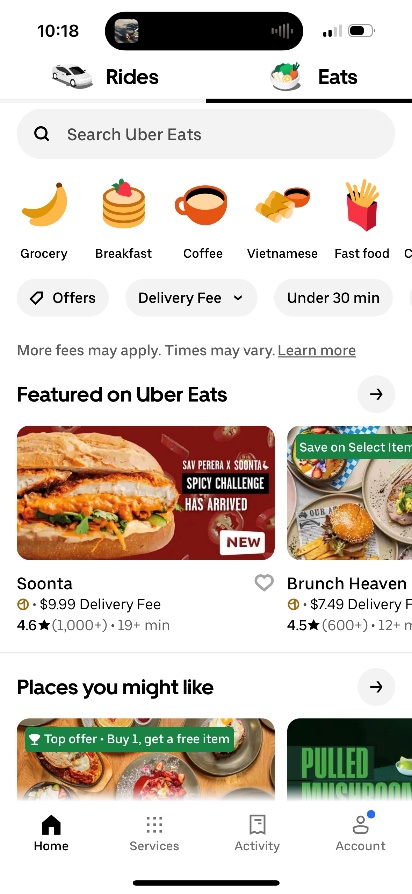 |
| Prioritised Item | 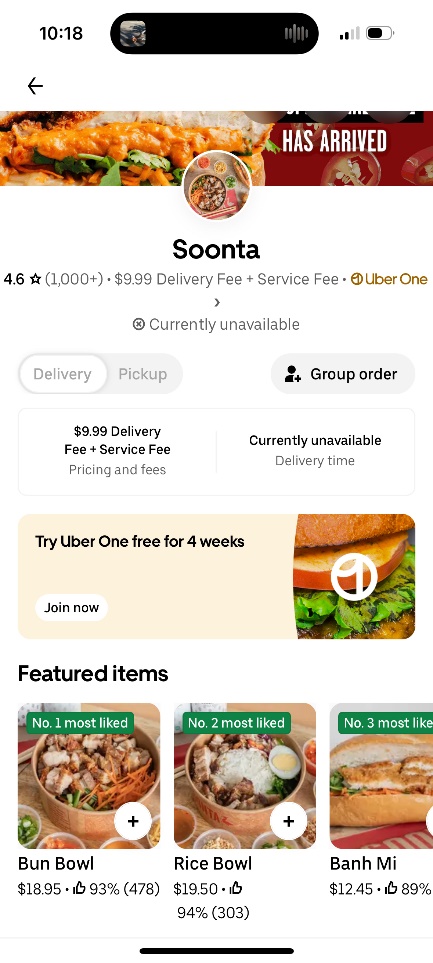 |
| Image | 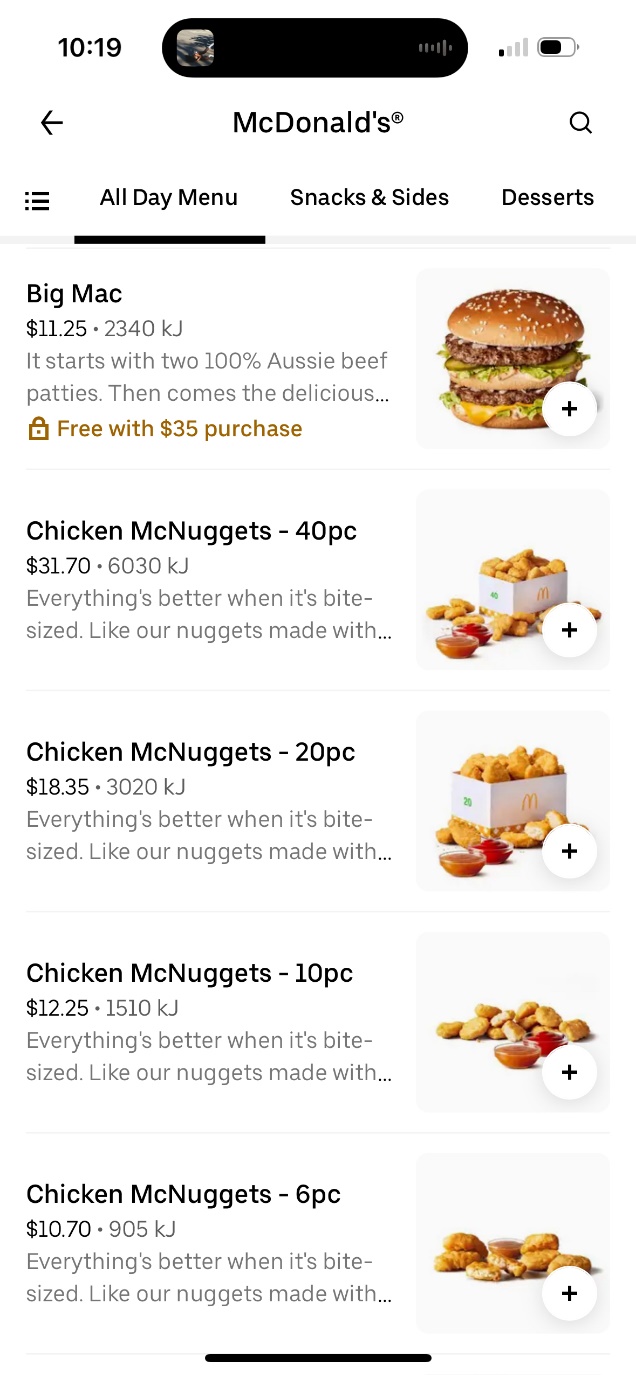 |
| Combination deal | 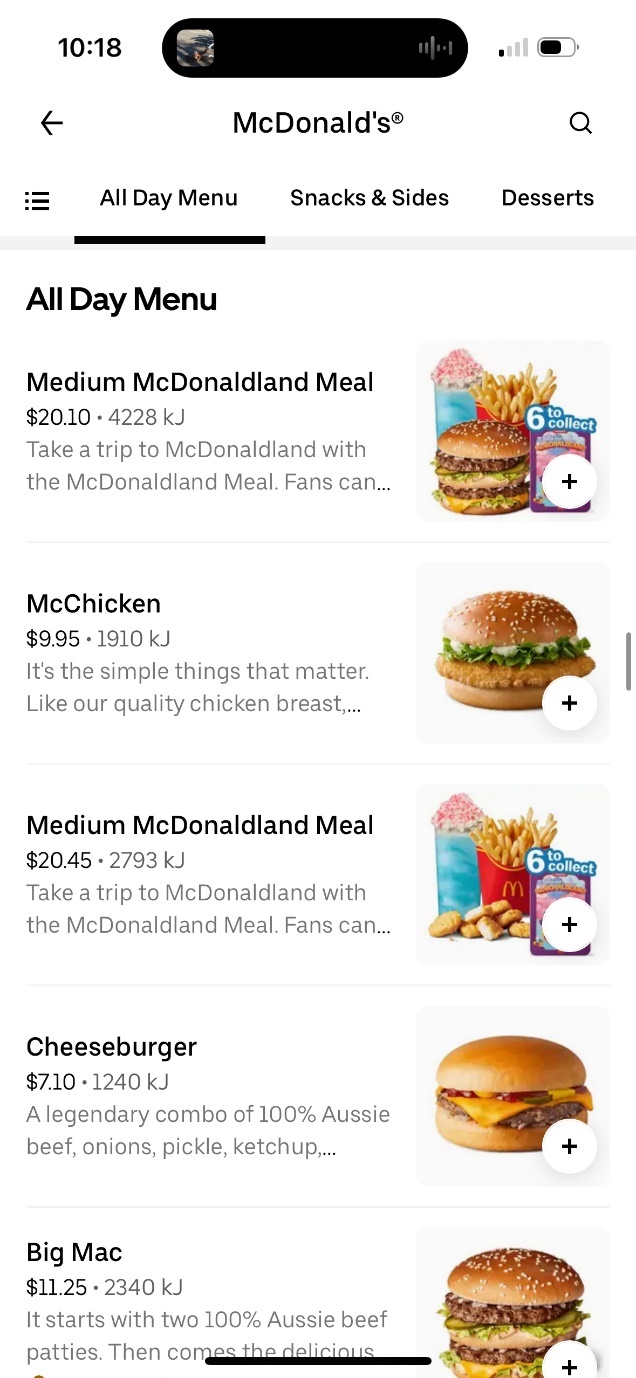 |
| Price promotion | 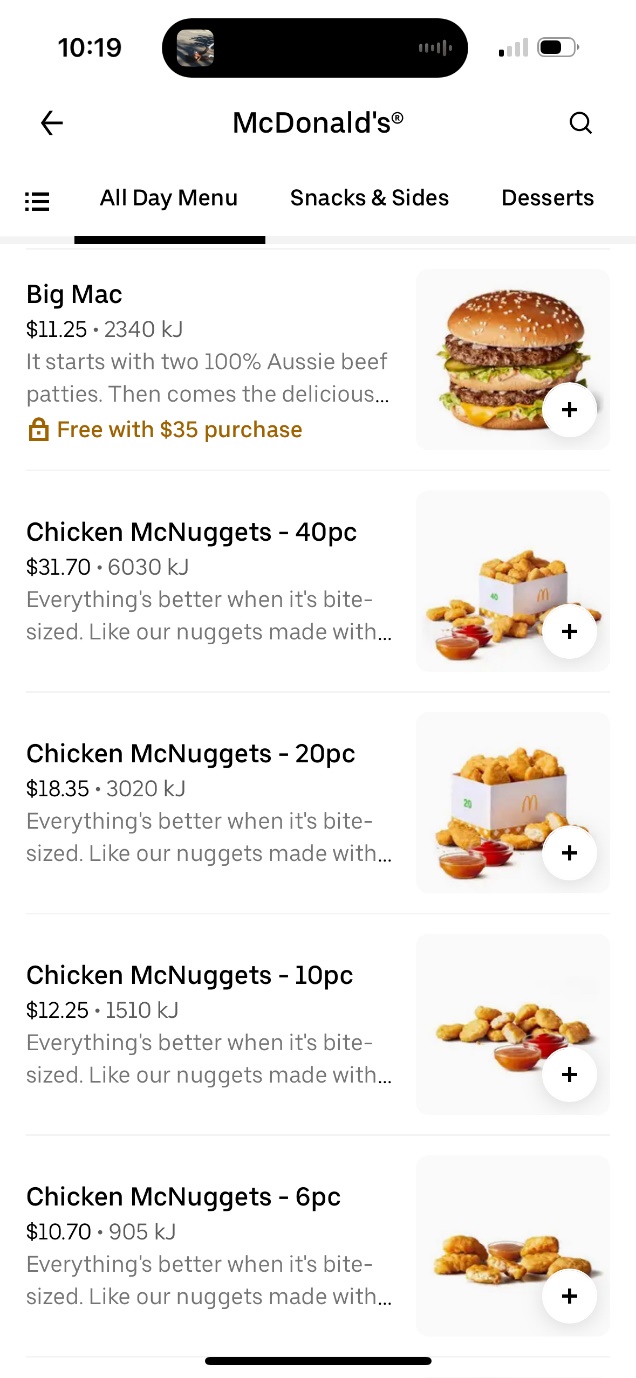 |
| Health claim | 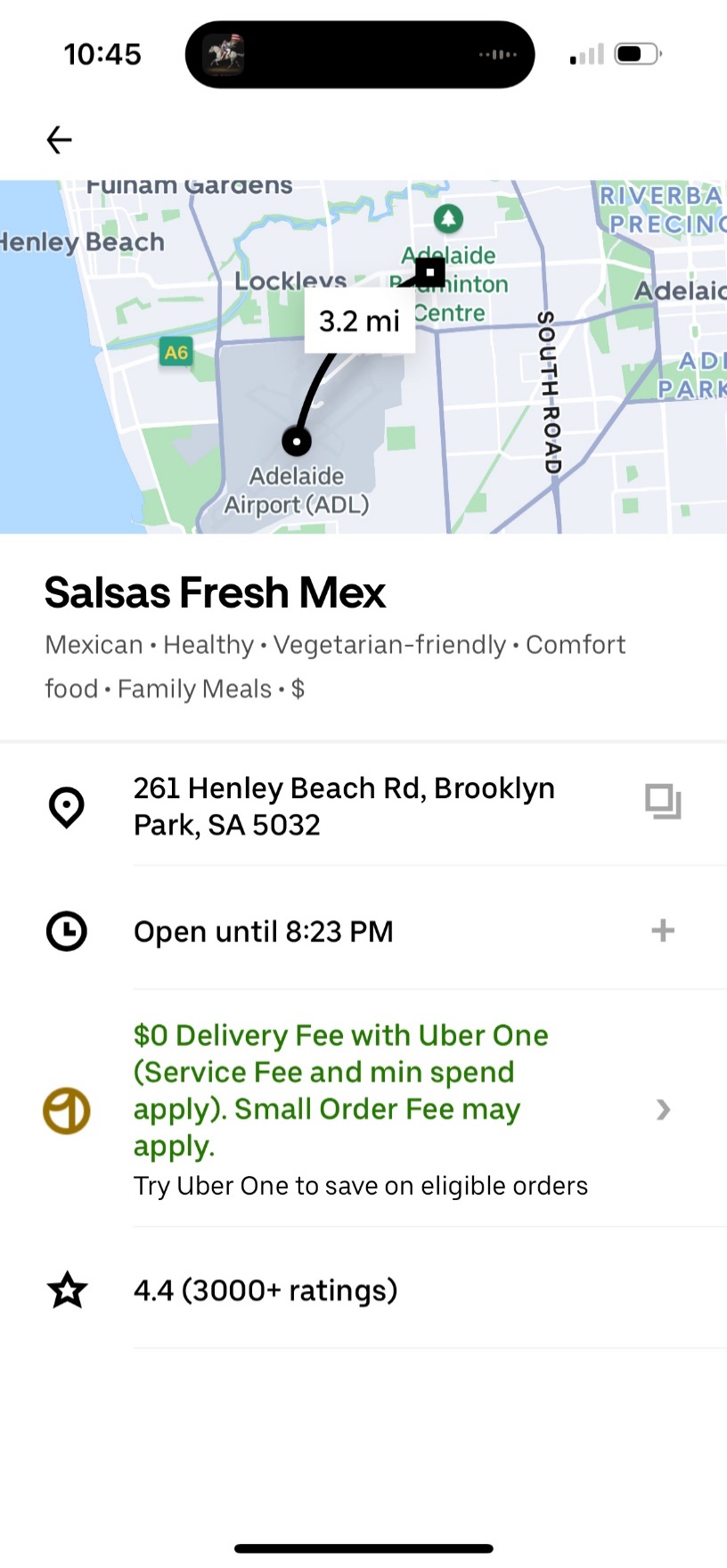 |
| Other claims | 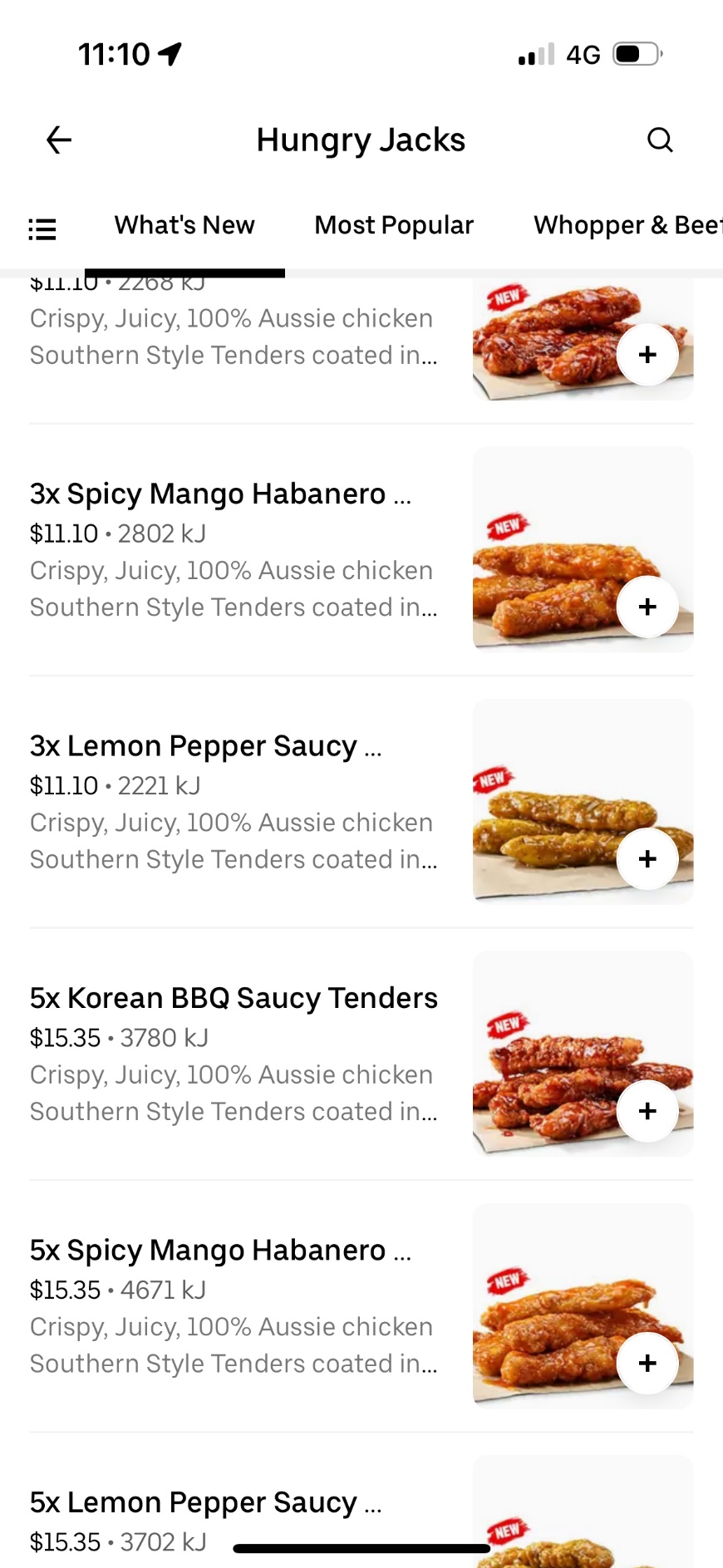 |

*Note.* Example images have been attained from the UberEats Australia (2025) website to illustrate the type of promotion and may not exactly match the graphical depictions captured from meal delivery apps assessed in the studies.
